# Determining promoter location based on DNA structure first-principles calculations

**DOI:** 10.1186/gb-2007-8-12-r263

**Published:** 2007-12-11

**Authors:** J Ramon Goñi, Alberto Pérez, David Torrents, Modesto Orozco

**Affiliations:** 1 Institute for Research in Biomedicine, Parc Científic de Barcelona, Josep Samitier, Barcelona 08028, Spain; 2 Departament de Bioquímica i Biología Molecular, Facultat de Biología, Avgda Diagonal, Barcelona 08028, Spain; 3 Grup de recerca en Bioinformàtica i Estadística Mèdica, Departament de Biologia de Sistemes, Universitat de Vic. Laura, 13 08500 VIC, Spain; 4 Computational Biology Program, Barcelona Supercomputer Center, Jordi Girona, Edifici Torre Girona, Barcelona 08028, Spain; 5 Institut Català per la Recerca i Estudis Avançats (ICREA), Passeig Lluís Companys, 23. Barcelona 08010, Spain; 6 Instituto Nacional de Bioinformática, Structural Bioinformatics Unit, Parc Cientific de Barcelona, Josep Samitier, Barcelona 08028, USA

## Abstract

A new method for the prediction of promoter regions based on atomic molecular dynamics simulations of small oligonucleotides has been developed. The method works independently of gene structure conservation and orthology and of the presence of detectable sequence features. Results obtained with our method confirm the existence of a hidden physical code that modulates genome expression.

## Abstract 2

A new method is presented which predicts promoter regions based on atomistic molecular dynamics simulations of small oligonucleotides, without requiring information on sequence conservation or features.

## Background

Sequencing projects have revealed the primary structure of the genomes of many eukaryotes, including that of human as well as other mammals. Unfortunately, limited experimental data exist on the detailed mechanisms controlling gene expression; this dearth of data has largely arisen from the difficulties found in the identification of regulatory regions. Traditionally, the immediate upstream region (200-500 bps) of a transcribed sequence is considered the proximal promoter area, where the binding of multiple transcription factor proteins triggers expression [1]. Other regulatory signals are found in distal regions (enhancers) that, despite being very far away in terms of sequence base pairs, can interact with the pre-initiation complex through the chromatin quaternary structure [1].

From a naïve perspective, the identification of promoter regions might be considered a trivial task, since they should be located immediately upstream (5') of the annotated transcribed regions. Unfortunately, the real situation is much more complex: on the one hand, 5' untranslated regions (UTRs) are very poorly described, and on the other, one gene might have several transcription start sties (TSSs) controlled by one or more proximal promoter regions (sometimes overlapping) scattered along gene loci, including introns, exons and 3' UTRs [2-6]. As a consequence, inspection of gene structure alone does not guarantee that the promoters will be located, and then, other signals need to be used to do this. Unfortunately such signals are very unspecific. Thus, transcription factor proteins are promiscuous and, depending on the genomic environment and the presence of alternative binding proteins, a given sequence can be recognized or ignored by the target protein. More general sequence signals also give noisy, unspecific signals. For example, the TATA box [7], which was originally believed to be associated with nearly all promoters, has been found to be present in only a small proportion of them [2,4]. A more powerful promoter signal stems from the presence of CpG islands [8-19], but even when present their signal is rather diffuse and unspecific. In summary, promoter detection is one of the greatest experimental and computational challenges in the post-genomic era.

Current methods for promoter location are based on two approaches: the use of gene structure and conservation; and the existence of sequence profiles that might signal promoter region. In the first case, statistical algorithms are used to find signals of genes that locate the 5'-end and conserved regions upstream [20]. For the second case, many sequence/compositional rules haven been used. Thus, several algorithms have been developed to detect signals like the TATA box, CpG islands or regions with large populations of transcription factor binding sites (TFBSs) [1,12,13,16,21-28]. Compositional rules (from trimer to n-mer) have also been considered to enrich the differential signal at promoters [1,12,13,21-28]. Finally, some methods have used predicted gene structure [1,12,21,22,27-29] and its conservation across species [1,28,29] to help their sequence-trained models to locate promoters. However, despite recent progress, the performance of all these methods is not great, especially when used to predict promoters that are not part of canonical 5' upstream regions [5,11,15,23].

Clearly, diffuse factors other than the specific hydrogen-bond interactions between nucleotides and binding proteins modulate the recognition of target DNA fragments in promoter regions. As first suggested by Pedersen *et al*. [30], one of these additional factors can be the physical properties of DNA, which control the modulation of chromatin structure, the transmission of information from enhancers or proximal promoters, and the formation of protein aggregates in the pre-initiation complex. Thus, Pedersen and others have shown how some descriptors that are believed to be related to physical characteristics of DNA (such as DNase I susceptibility, A-phylicity, nucleosome preference, DNA stability, and so on, up to 15 strongly correlated descriptors [31]) can help to locate promoters in prokaryotes and, perhaps, in eukaryotes [14,30,32-35]. Recent versions of progams like *mcpromoter *[33] or *fprom *[1] have incorporated these parameters into their predictive algorithms [1,5,33].

In this paper, for the first time, we explore the possibility of using a well-defined physically based description of DNA deformability [36] derived from atomic simulations to determine promoter location. Parameters describing the stiffness of DNA were rigorously derived from long atomistic molecular dynamics (MD) simulations in water using a recently developed force-field fitted to high level *ab initio *quantum mechanical calculations [37]. Using exclusively these simple parameters, whose interpretation is clear and unambiguous, we developed an extremely simple predictive algorithm which performs remarkably well in predicting human promoters, even those located in unexpected genomic positions.

## Results and discussion

### Derivation of stiffness parameters of DNA from molecular dynamics simulations

The use of a recently developed force-field [37] allowed us to perform long MD simulations (50 ns) of different DNA duplexes from which parameters describing dinucleotide flexibility can be obtained. Trajectories are stable with the DNA maintaining a B-type conformation with standard hydrogen bond pairings (Figures S1 and S2 in Additional data file 1), no backbone deformations [37,38], and normal distributions on helical parameters (Figures S3 and S4 in Additional data file 1) centered on expected values.

In contrast to assumptions in ideal rod models, DNA deformability is largely dependent on sequence. For example, it is possible to unwind (with the same energy cost) a d(CG) step twice than a d(AC) one (see Table 1). Our analysis shows also that some steps are universally flexible (like d(TA)), while others are, in general, rigid (like d(AC)). However, the concept of 'stiffness' associated with a step is often meaningless, since depending on the nature of the helical deformation, the relative rigidity of two steps can change (Table 1). In summary, flexibility appears as a subtle-sequence dependent process that is quite difficult to represent without the help of powerful techniques like MD simulations.

**Table 1:** Stiffness constants associated to helical deformations

Step	Twist	Tilt	Roll	Shift	Slide	Rise
AA	0.026	0.038	0.020	1.69	2.26	7.65
AC	0.036	0.038	0.023	1.32	3.03	8.93
AG	0.031	0.037	0.019	1.46	2.03	7.08
AT	0.033	0.036	0.022	1.03	3.83	9.07
CA	0.016	0.025	0.017	1.07	1.78	6.38
CC	0.026	0.042	0.019	1.43	1.65	8.04
CG	0.014	0.026	0.016	1.08	2.00	6.23
GA	0.025	0.038	0.020	1.32	1.93	8.56
GC	0.025	0.036	0.026	1.20	2.61	9.53
TA	0.017	0.018	0.016	0.72	1.20	6.23

Constants related to rotational parameters are in kcal/mol degree^2^, while those related to translations are in kcal/mol Å^2^.

### Differential physical properties of human promoters

**Figure 1:**
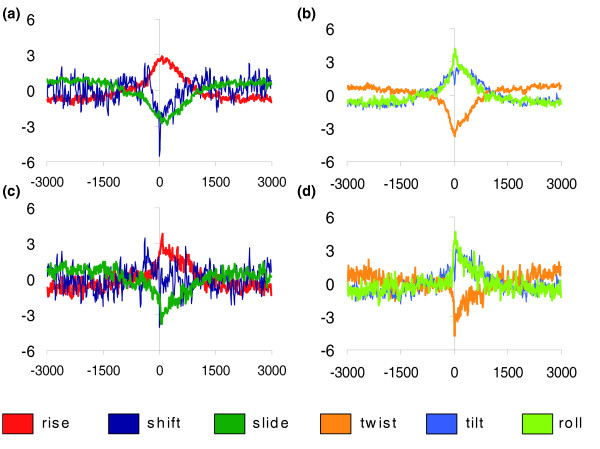
Measurement of the six 'average' helical force-constants. **(a,c) **Rise, shift, and slide; **(b,d) **twist, tilt, and roll. Results are shown for the complete training set of promoter regions (a,b) (see Materials and methods) and for the subset with no CpG island (c,d). Sequences are aligned at point +1 by its annotated TSS. All values are centered at zero (the background values).

From the analysis of helical stiffness along the human genome (see parameters in Table 1 and Materials and methods), we detected regions with distinctive structural properties that show a strong correlation with annotated TSSs (located using the 5' end of the human Havana gene collection [39] in the Encode region [40]). In particular, this signal was significantly stronger in regions located from -250 bp to +900 bp of the TSSs (that is, covering the core and proximal promoter regions; Figure 1), which agrees with the particular structural needs attributed to the correct function of regulatory regions. Interestingly, the differential signal found at the genome-scale does not appear to depend exclusively on the presence of CpG islands since the same signature is also present (even with less intensity) in promoters with standard CpG content (Figure 1c,d). Compared to those regions that are located far from annotated TSSs, the structural pattern measured for regulatory regions is quite complex: high flexibility near TSSs is required for some parameters, while rigidity is needed for others (Figure 1). Thus, our results suggest that the pattern of flexibility needed in promoter regions is quite unique, and general concepts like 'curvature propensity' or 'general flexibility' are too simplistic to capture the real average physical properties of promoter regions. We can speculate that the need for proper placement of nucleosomes, combined with the specific structural requirements of multi-protein complexes, favor the presence of sequences with unique deformation properties in the promoter region (especially in the core and proximal regions), which can be measured computationally.

### Using structural parameters for promoter prediction: ProStar

Taking advantage of the specific pattern of flexibility of promoter regions described above, we developed a new predictive algorithm called ProStar (for Promoter Structural Parameters; see Materials and methods), which uses only descriptors derived from physical first-principle type calculations (Table 1) to locate promoter regions (including strand orientation). Our method is conceptually and computationally simpler than any other general promoter prediction algorithm as it does not require any additional information, such as conservation of gene structure across species, presence of CpG islands, TATA-boxes, Inr elements or any other sequence specific signals. Due to its simplicity, ProStar can, in principle, be applied even in cases where promoters are located in unusual genomic positions.

**Figure 2:**
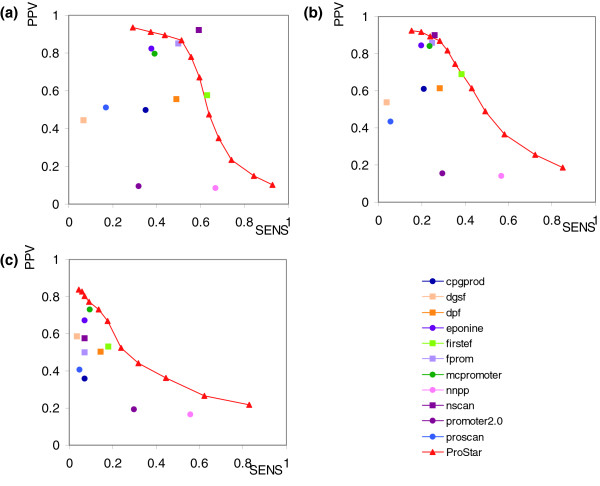
Results of performance comparison for the Encode region between ProStar and other programs (Table S1 in Additional data file 2) using a window size D equal to 1,000 (see Materials and methods). Results obtained compare the predictive power with **(a) **a subset of 885 Havana protein coding genes, **(b) **a set of 1,764 non-coding genes, and **(c) **a set of 1,086 annotated TSSs from a Cage data set that falls inside non-CpG island coding genes (see Materials and methods). Squares indicate methods based on gene prediction (exons, intronic signals, and so on), and other methods are represented with circles.

In order to evaluate the performance of our methodology in the context of other promoter predicting approaches (see Materials and methods and Table S1 in Additional data file 2), we compared our results with those derived from other reported promoter predictors, following the Egasp workshop procedures [5,41] and using the annotation of the Havana team [39] for the Encode regions [40] as the reference set. In order to cover the whole spectrum of prediction methodologies, we selected a few representative procedures mainly based on the conservation of gene structure (*fprom *[1], *firstef *[13], *dpf *[12] and *nscan *[29]), the identification of CpG islands (*eponine *[22], *cpgprod *[16] and *dgsf *[21]), compositional sequence biases (*mcpromoter *[26,33]) and other criteria (*nnpp *[24] and *promoter2.0 *[25]). The results of these comparisons show that despite its simplicity, ProStar performed better than most of the other methods and was similar to two algorithms that use gene structure for prediction (*fpom *and *firstef*), and only *nscan*, which is based also on multi-species homology, provided more accurate results for the reference set of genes (Figure 2, Table 2 and Figure S5 in Additional data file 1). Global analysis of performance using Bajic's metrics [42] (see Materials and methods) showed that the predictive power of our method is only improved by *nscan *(Table 2 and Table S2 in Additional data file 2). Furthermore, when the calculations used to derive the results shown in Figure 2 are repeated using a more restrictive tolerance test (window size D = 250; see Materials and methods), the superiority of ProSart with respect to most of the other methods was maintained (Figure S6 in Additional data file 1) in most regions of a 'proportion of correct predictions (PPV)/sensitivity (SENS)' map, demonstrating the robustness of our method. Finally, it is worth to comment the good performance of ProStar, that only uses simple dinucleotide parameters, compared to complex methods based on n-mer compositional rules (see Materials and methods). Clearly, the richness of the six-dimensional descriptors obtained for each dinucleotide by the MD simulation explains the success of our simple approach.

**Table 2:** Global ASM performance index obtained by considering Bajic's muti-metric analysis for different sets of genes

	CDS_gene	no_CDS_gene	noCpG	no_CpG_CAGE
ProStar	2.78	**2.00**	6.56	**2.56**
cpgprod	8.22	7.89	7.22	7.11
dgsf	9.56	9.11	9.11	7.00
dpf	6.78	7.00	4.89	5.67
eponine	5.56	6.11	8.33	3.78
firstef	4.00	4.00	5.56	3.78
fprom	3.56	3.22	2.78	9.78
mcpromoter	5.56	5.33	4.89	4.89
nnpp	10.44	10.33	9.44	8.89
nscan	**1.56**	2.89	**1.22**	6.89
promoter2.0	10.67	10.56	8.89	9.33
proscan	9.33	9.56	9.11	8.33

Global ASM performance index obtained following Bajic's muti-metric analysis (see Materials and methods) for different sets of genes: the 2,641 TSSs from the Havana set (column CDS_gene), the 1,764 TSSs of non-coding genes from the Havana set (column no_CDS_gene), the 1,751 TSSs of the Havana set that do not overlap any CpG island (column noCpG), and the collection of 1,086 Cage TSSs not associated with CpG islands (no_CpG_CAGE). In each case the method providing the best results is shown in bold. Note that ProStar is the best in the two most difficult categories and the second best over the entire set of genes.

Interestingly, when the analysis is performed for a subset of TSSs of non-coding genes (Figure 2, Table 2 and Figure S6 in Additional data file 1) the performance of all the methods decreases, but ProStar seems more robust than the others. In fact, the analysis of these data shows that, for this subset of genes, ProStar performs better than any method that uses sequence compositional bias, location of known TFBSs, or the presence of TATA-box signals or CpG islands and similar or better than those relying on the presence of orthologs as shown in Bajic's metrics (Table 2).

### Testing ProStar against non-trivially identified promoters

Our method works better when predicting promoters associated with CpG islands, but the decrease in performance for promoters associated with non-CpG islands is similar to that of other methods, including those that are based on the maintenance of the gene structure (Figure S7a in Additional data file 1). If a conservative definition of a non-CpG associated promoter is used (no CpG island detectable at less than 5 Kb from the promoter), the performance of ProStar decreases, but is still better than that of most methods (Figure S7b in Additional data file 1), although even in this case the method is not competitive with algorithms based on gene structure conservation. In any case the performance of ProStar for genes not associated with CpG islands is quite reasonable, confirming that the need for specific elastic properties at promoter regions is a general requirement and not restricted to the presence of CpG islands or diffuse TSSs. It is also worth noting that ProStar performs better than methods specifically tuned to capture promoters associated with CpG islands when the analysis is restricted to Havana annotated genes with CpG islands (data not shown). Finally, the performance of ProStar does not decay for genes containing a TATA box (Figure S8 in Additional data file 1), which are the easiest to detect from simple sequence signals.

Once we tested the performance of ProStar to reproduce promoters annotated by the Havana group, we explored the ability of the method to locate promoters reported in massive Cage experiments [4], where promoters were often found in unexpected locations. To increase the challenge, we analyzed only Cage-detected promoters falling inside transcribed regions (including exons and 3' UTR regions) of annotated Havana genes that are not regulated by a CpG island. Our results demonstrate that despite the method not being trained with this type of promoter, it performed quite well (Figure 2, Table 2, Figures S6 and S9 in Additional data file 1), in fact improving the results obtained by other available methods (Table 2).

ProStar calculations were repeated throughout the entire human genome using TSS positions according to RefSeq genes. The results are summarized in Figure S10 in Additional data file 1 and confirm the quality of our predictions at the genome level. Please note that some caution is needed in the interpretation of these results since the apparent better performance of our method at the genome level compared with that obtained using Encode regions can be simply due to the noise in the first dataset.

**Figure 3:**
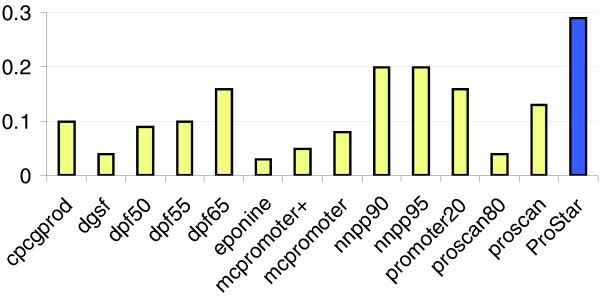
CC measurement (see Materials and methods) for the subset of Havana TSSs (1,203) of non-coding protein genes in the Encode region, unrecalled by *nscan*, *fprom *and *firstef*.

The final extreme challenge for ProStar was to find promoters that are not detectable by methods based on sequence conservation along orthologs or on the maintenance of gene structure. For this purpose, we selected a subset of 1,203 annotated promoters of non-coding genes that are found as false negative by *nscan*, *fprom *and *firstef*. We should clarify that this comparison will give no information on ProStar with respect to 'state of the art' methods based on conservation of gene structure and orthology, but does give some indication of the ability of other methods (including ProStar) to capture promoters located in anomalous positions. The results shown in Figure 3 demonstrate that ProStar can recover a significant fraction of these promoters with a signal to noise ratio superior to all methods based on the differential genomic content of promoters and on the use of powerful discriminant algorithms. This suggests that ProStar is a powerful tool for promoter determination and that it could be a good alternative for the location of promoters of fast evolving genes or those appearing in anomalous positions that violate the traditional concept of gene structure.

## Conclusion

Atomic MD simulations, based on physical potentials derived from quantum chemical calculations, yield helical stiffness parameters that reveal the complexity of the deformation pattern of DNA. The use of these intuitive parameters at the genomic level allowed us to define promoters as regions of unique deformation properties, particularly near TSSs. Taking advantage of this differential pattern, we trained a very simple method, based on Mahalanobis metrics, that is able to locate human promoters with remarkable accuracy. Our results are better than the ones of methods based on the use of large batteries of descriptors, such as sequence signals, empirical physical descriptors, and complex statistical predictors (neural networks, hidden Markov models, and so on). The overall performance of ProStar is similar and in some cases even better than that of methods based on the conservation of gene structure, methods that might not be so accurate in the location of promoters of fast evolving genes, or those located in unusual positions. Taken together, our work reveals that even in complex organisms like human, there is a hidden physical code that contributes to the modulation of gene expression.

## Materials and methods

### Molecular dynamics simulations

In order to have enough equilibrium samplings for all the ten unique steps of DNA, we performed MD simulations of four duplexes containing several replicas of every type of base step dimer (d(GG), d(GA), d(GC), d(GT), d(AA), d(AG), d(AT), d(TA), d(TG) and d(CG)): d(GCCTATAAACGCCTATAA), d(CTAGGTGGATGACTCATT), d(CACGGAACCGGTTCCGTG) and d(GGCGCGCACCACGCGCGG). All duplexes were created in the standard B-type conformation, hydrated with around 10,600 water molecules, and neutralized by adding a suitable number of Na^+ ^ions. Neutral hydrated systems were then optimized, thermalized and pre-equilibrated using our standard protocol [43,44]. The structures obtained at the end of this procedure were then re-equilibrated for an additional 2 ns. The snapshots obtained at the end of this equilibration were used as starting points for 50 ns trajectories performed at constant temperature (298 K) and pressure (1 atm) using periodic boundary conditions and Ewald summations [45]. Simulations were carried out using SHAKE [46] on all bonds connecting hydrogens and 2 fts time steps for integration of Newton equations of motions. TIP3P [47] was used to represent water, while PARMBSC0 [37,48,49] was used to represent DNA.

Trajectories were manipulated to obtain the stiffness matrix (Ξ; equation 1) representing the deformability of a given step along rotations (twist, roll and tilt) and translations (rise, slide and shift) from equilibrium values. For this purpose we determined the oscillations of all these parameters, building a covariance matrix whose inversion led to the stiffness matrix (equation 1) [36,50-53], which is simplified for each dinucleotide step as a six-dimensional vector *κ *= (*k*_*twist*_, *k*_*roll*_, *k*_*tilt*_, *k*_*rise*_, *k*_*shift*_, *k*_*slide*_) by neglecting the out-of-diagonal terms in the stiffness matrix (equation 1). Note that each of these elements (*k*_*i*_) is the force-constant associated with the distortion along a given helical coordinate:

$$\Xi ={\left(k_{B}T\right)}^{-1}•C_{h}^{-1}=\left[\begin{array}{cccccc} k_{twist} & k_{t-r} & k_{t-l} & k_{t-i} & k_{t-s} & k_{t-d}\\ k_{t-r} & k_{roll} & k_{r-l} & k_{r-i} & k_{r-s} & k_{r-d}\\ k_{t-l} & k_{r-l} & k_{tilt} & k_{l-i} & k_{l-s} & k_{l-d}\\ k_{t-i} & k_{r-i} & k_{l-i} & k_{rise} & k_{i-s} & k_{i-d}\\ k_{t-s} & k_{r-s} & k_{l-s} & k_{i-s} & k_{shift} & k_{s-d}\\ k_{t-d} & k_{r-d} & k_{l-d} & k_{i-d} & k_{s-d} & k_{slide} \end{array}\right]$$

where *k*_*B *_is Boltzman's constant, T is the absolute temperature and *C*_*h *_is the covariance matrix in helicoidal space (for a given base step pair) obtained from the MD samplings.

### Datasets

ProStar was trained using 5' ends of protein coding genes annotated by the Havana group [39] in the human Encode [40] region as a TSS set. According to Egasp workshop rules [5], the training procedure was restricted to 13 of the 44 Encode regions (see performance test section). TSS and strand recognition are trained and processed independently. ProStar requires a sequence with a minimum length of 500 nucleotides for TSS identification (see TSS prediction section). This size is extended to 1,800 nucleotides for strand prediction (see Strand prediction section).

Encode regions and annotated data and predictions were downloaded from the Egasp ftp directory [54]. We used *version00.3_20may *[55] of the Havana annotation and 'submitted_predictions' of the *egasp_submissions_20050503 *directory [56] as predicted TSSs (Table S1 in Additional data file 2). The number of Havana TSSs that fall inside the Encode region is 2,641, but only 885 (34%) are coding genes. Coding genes are those with annotated start and stop codon signals; the others are taken as non-coding.

In addition to Egasp test sets, we analyzed the performance of our methodology using the selected sets of TSSs more difficult to predict (as TSSs on unexpected positions or TSSs belonging to genes with special particularities). These sets are a particular subset of 1,764 TSSs of Havana annotated non-coding genes (67% of Havana TSSs), 1,751 TSSs of coding and non-conding genes without upstream CpG islands (66% of the Havana set), 2,255 TTSs missing a TATA-box (85%), and the 1,086 unexpected TSSs positioned inside introns or exons of coding genes without CpG islands, as found in Cage predictions. CpG islands were mapped according to the UCSC database [57,58]. Since CpG islands are supposed to be the strongest promoter signals, this set represents an important challenge for our method. TATA-boxes were scanned in the proximal 50 nucleotide upstream region relative to the TSS, using the TATA position weight matrix [59] and the standard cut-off (-8.16). Cage predictions [60] were downloaded from Egasp [54] database. Those overlapping any Havana coding and non-coding genes (without a CpG island in the upstream region) were selected. Standard Egasp rules were used also for these challenging sets.

### Training

We trained our method for promoter recognition with a collection of 500-nucleotide sequences that comprised intervals of 250 nucleotides upstream and downstream of the training TSS set. As negative set, we collected 500-nucleotide sequences from transcribed regions of Havana coding genes. We made sure that positive and negative sequences did not overlap. For the recognition of the strand, we trained our method with a collection of DNA sequences that comprised (for every TSS in the positive training set) the 1,800 nucleotide DNA sequence ranging from 900 bp upstream to 900 bp downstream of the same TSS. The reverse complementary sequences of the positive set were taken as a negative set.

### Computation of DNA physical properties

Using our MD derived parameters (see Molecular dynamics simulations section and Table 1), we can describe any DNA sequence of size *n *as a six-dimensional deformation vector *v *= (*twist*, *tilt*, *roll*, *shift*, *slide*, *rise*). For a given deformation we sum the values associated with every dinuecleotide step in the sequence and divide the total by *n *- 1. For example, the *twist *deformation score for the sequence ACGC would be (0.036 [AC] + 0.014 [CG] + 0.025 [GC])/3 = 0.025. The six-dimensional vector of the same sequence would then be *v*(ACGT) = (0.025, 0.033, 0.022, 1.200, 2.547, 8.230).

### Transcription start site prediction

We used Mahalanobis distance [61] to classify 500-nucleotide DNA sequences as belonging to the promoter class (*k*_*x*_) or non-promoter class (*k*_*y*_). Every class is defined by a specific dataset of sequences (see Training set section). Computing the physical properties of every sequence of the dataset, we conclude with a set of vectors for every class (*X *for class *k*_*x *_and *Y *for *k*_*y*_). The Mahalanobis distance *D*_*M *_between the set of vectors *X *and *Y *is defined as:

*D*_*M*_(*X*, *Y*) = (*μ*_*x *_- *μ*_*y*_)^*t *^*C*^-1^(*μ*_*x *_- *μ*_*y*_)

where *μ*_*x *_and *μ*_*y *_are the average vectors of the sets *X *and *Y *and *C*^-1 ^is the covariance matrix of *X*U*Y*. The decision function *g *of a specific 500-nucleotide DNA sequence with a descriptor vector *s *to a class *k*_*i *_(with *i *= <*x*, *y*>) is defined as:

$$g(s,k_{i})=w_{k_{i}}^{t}s+w_{k_{i},0}$$

where $w_{ki}=C^{-1}\mu_{i};w_{k_{i},0}=-0.5\mu_{i}^{t}C^{-1}\mu_{i}$. When *g*(*s*, *k*_*x*_) > *g*(*s*, *k*_*y*_) we should classify our sequence as a promoter. Even so, we can modulate the confidence of our decision according to a normalized score defined in equation 4. If the score is greater than a specific threshold (set to +1 by default), then the sequence is flagged as a promoter.

$$score(s)=\frac{g(s,k_{x})-g(s,k_{y})}{g(\mu_{x},k_{x})-g(\mu_{x},k_{y})}$$

### Strand prediction

ProStar has been trained to recognize upstream/downstream signal asymmetry of predicted TSSs using a statistical discriminator based on Mahalanobis metrics (see last section) and on the differences in physical properties between the 0→-900 nucleotide and the 0→+900 nucleotide regions. The ProStar strand recognition module was trained using 1,800-nucleotide sequences with a TSS in the +900 position as the positive set. The reverse complement of the positive set sequences was used as the negative set.

### Prediction clustering

As observed using experimental approaches [4], TSSs have a dominant position, but many closely related alternative sites may be found around them. In consequence, every TSS may produce multiple close predictions. To clarify the annotation, our algorithm allows the user to define a window size (set as 1,000 nucleotides by default) where all predictions will be unified in a single annotation. Accordingly, for a given window *W *of a specific strand *q*, we define *P*(*W*, *q*), the set of positions *p *falling inside *W *with *score*(*p*, *q*) ≥ *c *(where *c *is the user-defined minimal cutoff). Predicted dominant position *p' *of the window *W *is computed as:

$$p'=\frac{\sum_{p}p\cdot score{\left(p,q\right)}^{2}}{\sum_{p}score{\left(p,q\right)}^{2}}$$

### Performance test

The training and performance of ProStar followed the protocol described [5] for the Egasp workshop [54,56]. Thus, protein coding genes annotated by the Havana group from 13 of the Encode regions were used for training, while the entire set was used in tests (tests performed using only regions that were not considered in the training give very close results; Table S2 in Additional data file 2). Also following the Egasp rules, true positives (TPs) are considered when the predicted TSS is in the same strand and at a maximum distance of D nucleotides from the annotated TSS (as in Egasp, D = 1,000 or D = 250 is used here). If the annotated TSS is missed using this criteria, we label the prediction as a false negative (FN). Every other prediction falling on the annotated part of the gene loci in the segment [+D+1, EndOfTheGene] counts as a false positive (FP). A true negative (TN) is the sum of positions falling on the gene loci segment [+D+1, EndOfTheGene] that do not overlap accepted true positive positions or any false positive prediction.

Sensitivity (SENS), proportion of correct predictions (PPV) and correlation coefficient (CC) are computed as:

$$SENS=\frac{TP}{TP+FN}$$

$$PPV=\frac{TP}{TP+FP}$$

$$CC=\frac{\left(TP\times TN)-(FP\times FN\right)}{\sqrt{\left(TP+FP)(TF+FN)(TN+FP)(TN+FN\right)}}$$

In addition to the standard performance measures noted above, we also consider the average mismatch of predictions (AE) [5] and other extended metrics suggested by Bajic [42], including specificity (SPEC), Yule's association coefficient (Q), second prediction quality coefficient (K2), and generalized distances from ideal predictors (GDIP1, GDIP2, GDIP3). We also include in our analysis the averaged score measure (ASM), which combines many 'independent' descriptors to provide an overall relative measure of the quality of a predictive method with respect to others (Table S2 in Additional data file 2; Additional data file 3).

In addition to the methods checked in the Egasp experiment, we performed predictions using programs that were not considered in the Egasp experiment, but which are publicly available. In these cases we used the corresponding web-based tool or downloadable script with default parameters (Table S1 in Additional data file 2). When possible, we modified these default parameters in the input to obtain PPV/SENS curves (see Results and Figure S6 in Additional data file 1) instead of a single prediction. All methods were evaluated following the same thresholds for annotation of positive and negative predictions (see above).

### Web server

ProStar is developed in C and compiled on a Linux machine. An unrestricted user-friendly version of the program is publicly available through our web server [62]. Strand prediction of recognized TSSs is an optional feature. Goodness of predictions may be tuned using a threshold (set to 1.0 by default) that may be increased to improve the proportion of correct predictions or decreased for sensitivity. Finally, the user may choice cluster size (see Prediction clustering section), which is set to 1,000 by default. Clustering may be avoided by setting this size to small values (for example, 1).

## Abbreviations

ASM, averaged score measure; CC, correlation coefficient; FN, false negative; FP, false positive; MD, molecular dynamics; PPV, proportion of correct predictions; SENS, sensitivity; SPEC, specificity; TFBS, transcription factor binding sites; TN, true negative; TP, true positive; TSS, transcription start sties; UTR, untranslated regions.

## Authors' contributions

RG developed the predictive code and trained the method. AP performed the MD simulations and obtained the stiffness parameters. DT was involved in the design of the experiments and discussion of results and corrected the manuscript. MO conceived and developed the idea, designed and discussed experiments and wrote the manuscript.

## Additional data files

The following additional data are available with the online version of this paper. Additional data file 1 provides supplementary figures showing plots of dinucleotide helical parameters and additional performance tests of ProStar. Additional data file 2 contains a list of promoter prediction methods described in this paper and a detailed evaluation of their performance. Additional data file 3 extends the description of the performance test and explains the averaged score measure (ASM)

## Supplementary Material

Additional file 1 Supplementary figures showing plots of dinucleotide helical parameters and additional performance tests of ProStar. Click here for file

Additional file 2 Promoter prediction methods described in this paper and a detailed evaluation of their performance. Click here for file

Additional file 3 Extended description of the performance test and the averaged score measure. Click here for file
