# Supplementary material for: Determining promoter location based on DNA structure first-principles calculations
Source: Genome Biol. 2007 Dec 11;8(12):R263. doi: 10.1186/gb-2007-8-12-r263 (PMC2246265; doi:10.1186/gb-2007-8-12-r263)
Supplement: Additional file 1 — Supplementary figures showing plots of dinucleotide helical parameters and additional performance tests of ProStar. [file gb-2007-8-12-r263-S1.pdf]

FIGURE S1

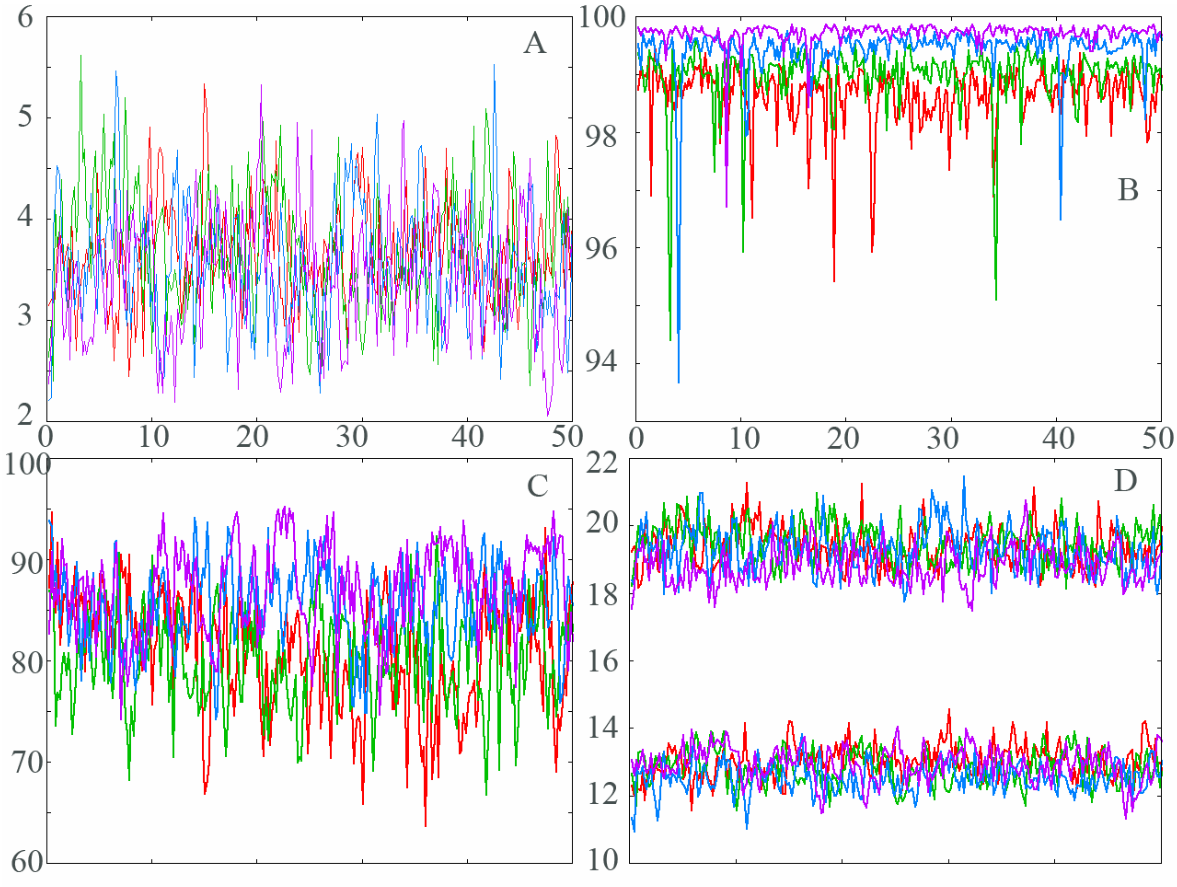

Figure 1. Smoothed control parameters along time (ns), ends were removed to avoid end effects. (A) rmsd (in Å) with respect to canonical B-DNA. (B) Percentage of conserved canonical H-bonds. (C) Percentage of south conformation. (D) Major and minor groove width (in Å) (phosphate radii not removed). Red: seq1,green: seq2,blue: seq3,purple: seq4.

FIGURE S2

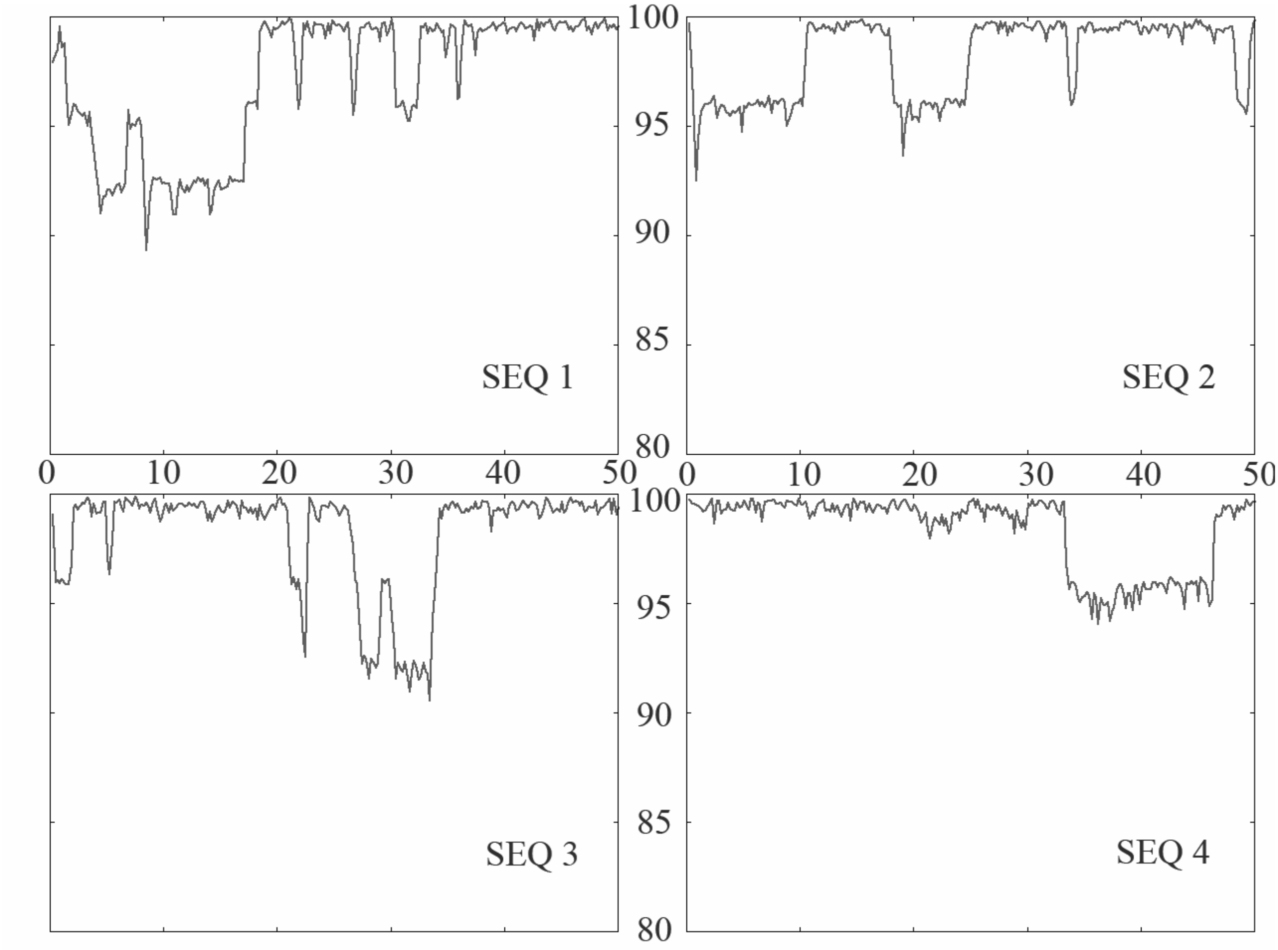

Figure S2. Smoothed percentage of total number of canonical alfa/gamma states along time (ns). As can be seen all transitions to non-canonical states happen in a reversible way. Terminal bases were removed from the analysis to avoid end effects.

FIGURE S3

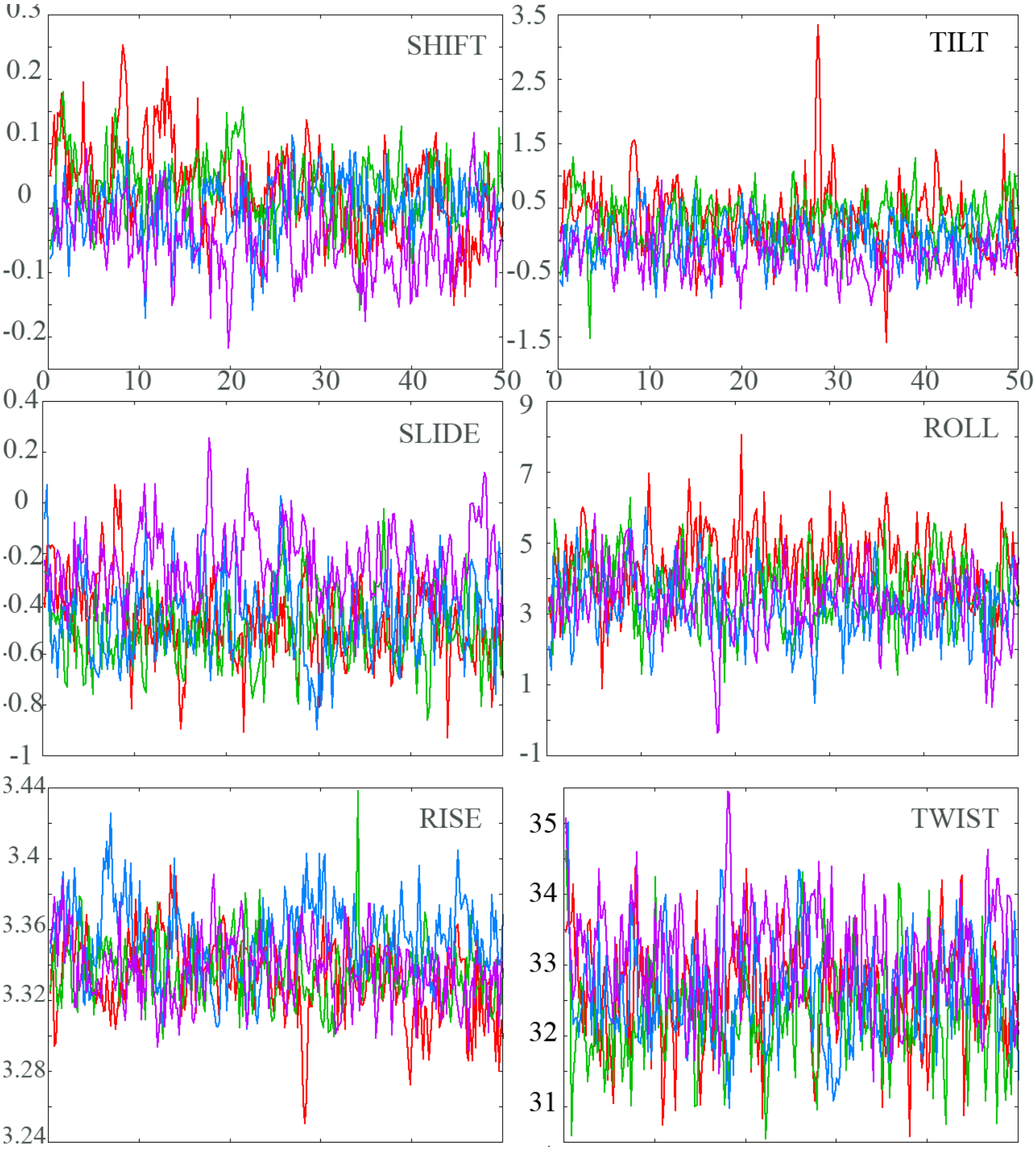

Figure S3. Base pair parameters along time (ns). Shift, slide and rise are in Å. Twist, tilt and roll in degrees. Each parameter represents the average of all base pair steps in the sequence. Terminal bases were removed from the analysis.

FIGURE S4

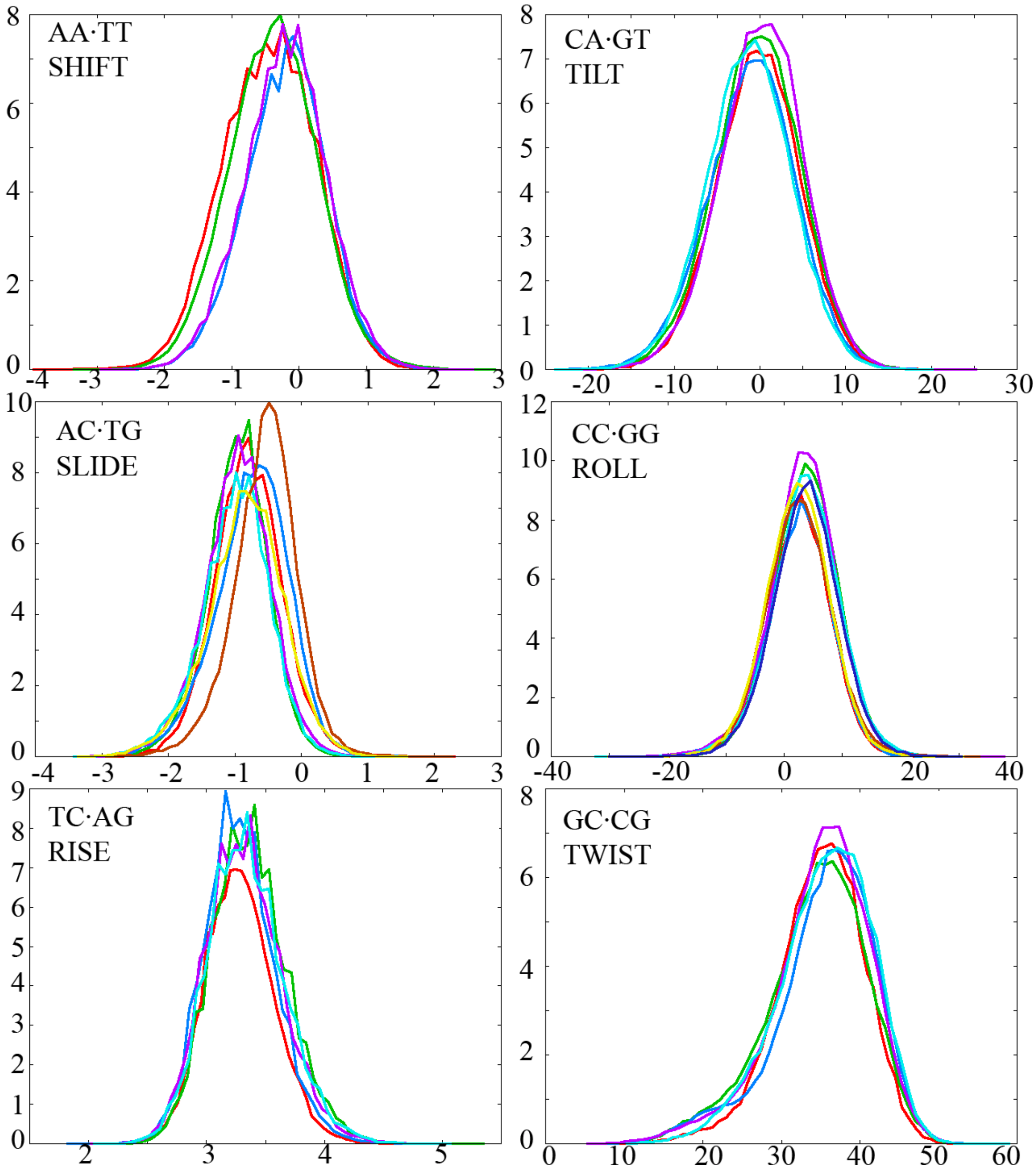

Figure S4. Some examples of distributions of local base pair parameters during trajectories. Different lines correspond to the behaviour of same base pair step found in the 4 duplexes. Analysis was performed removing the ending base pair steeps to avoid end effects. Note that the Gaussian shape of the population profiles justify the use of an harmonic deformation model for computing deformability.

FIGURE S5

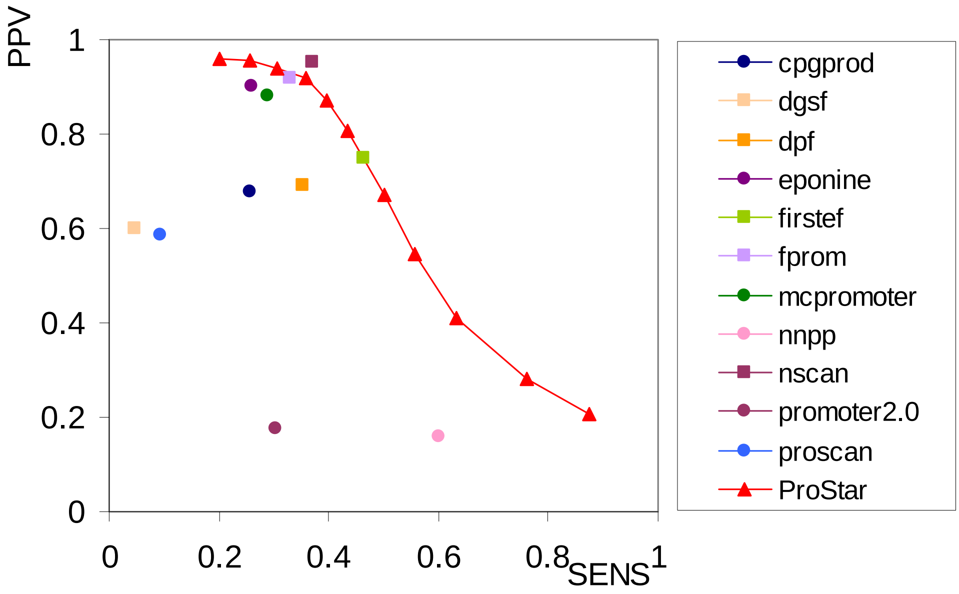

Figure S5. Test of ProStar behavior with the whole set (2641) of Havana (coding and non-coding) genes using tolerance D = 1000.

FIGURE S6

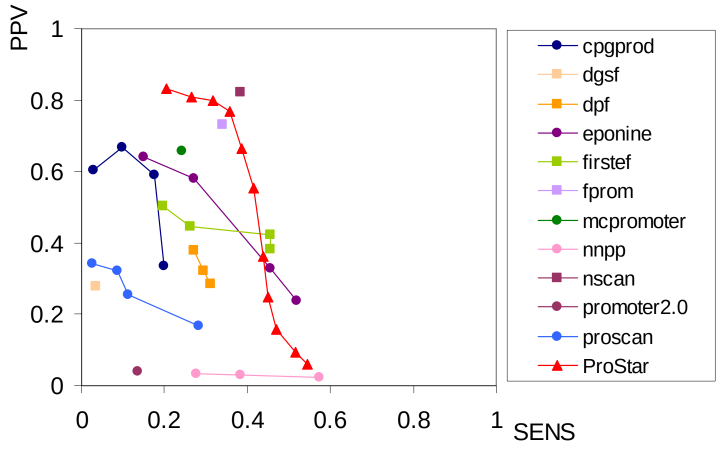

(A)

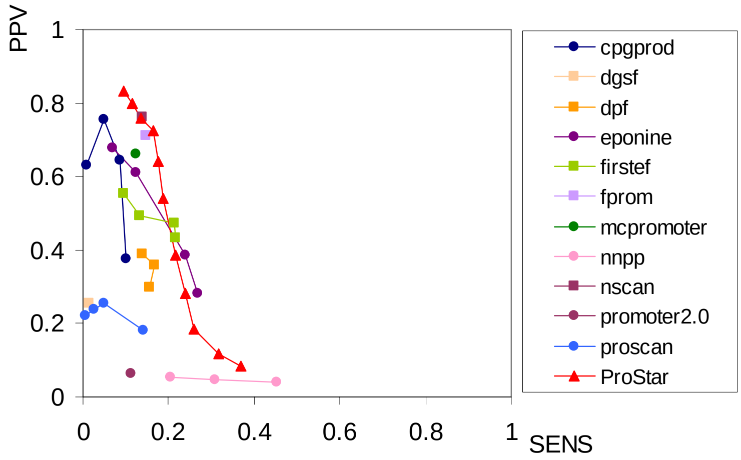

(B)

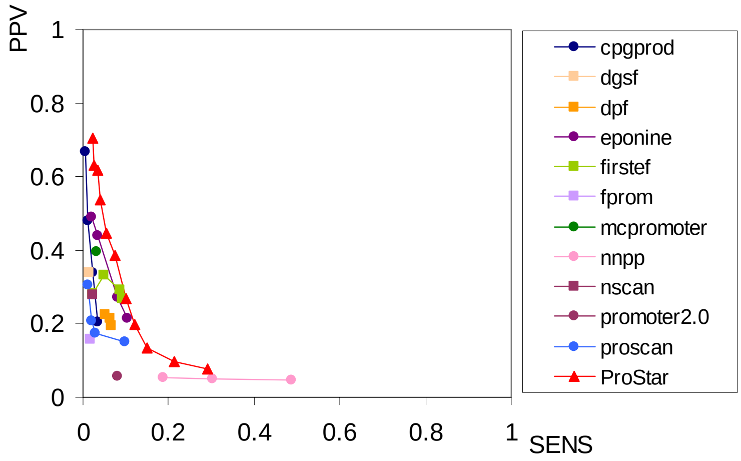

(C)

Figure S6. Results of performance comparison in the Encode region between ProStar and other programs using tolerance D = 250. (A) Results obtained comparing predictive power over a subset of 885 Havana protein coding genes, (B) a set of 1764 non-coding genes and (C) a set of 1086 annotated TSS from Cage data set that falls inside non-CpG island coding genes (see methods). When it was possible, SENS/PPV performance of the different methods were tuned using input parameters (threshold, cutoff, etc.) to obtain different PPV/SENS measures.

FIGURE S7

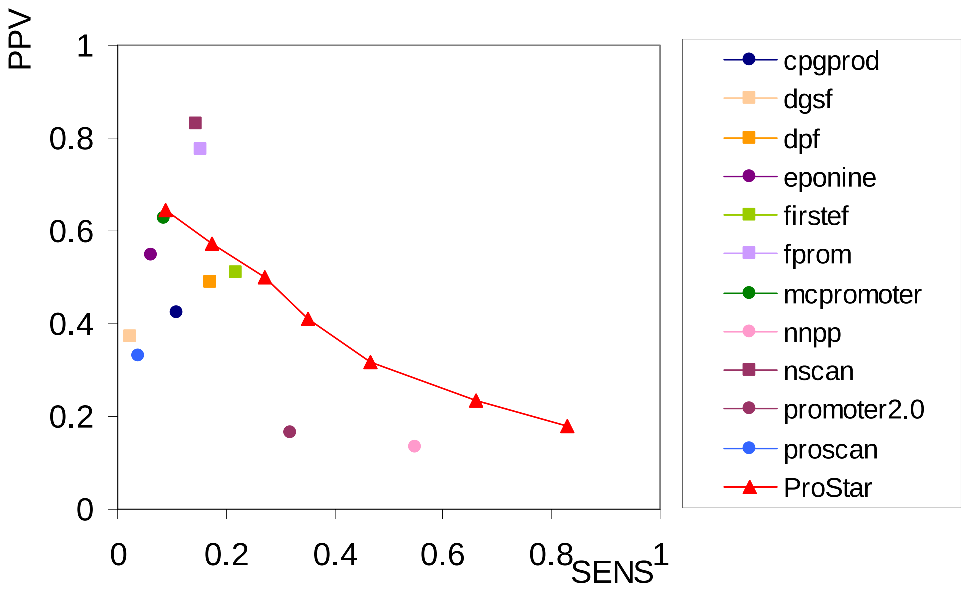

(A)

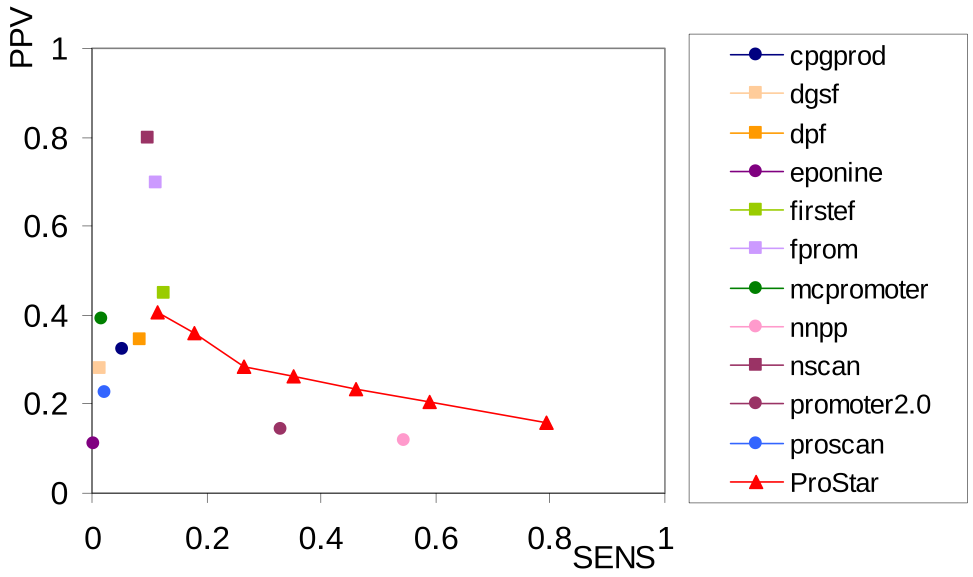

(B)

Figure S7. Test of ProStar behavior using those genes from the HAVANA set using tolerance D = 1000 with (A) no CpG island in the TSS (1751) and (B) no CpG island 5Kb away from the TSS (1209).

FIGURE S8

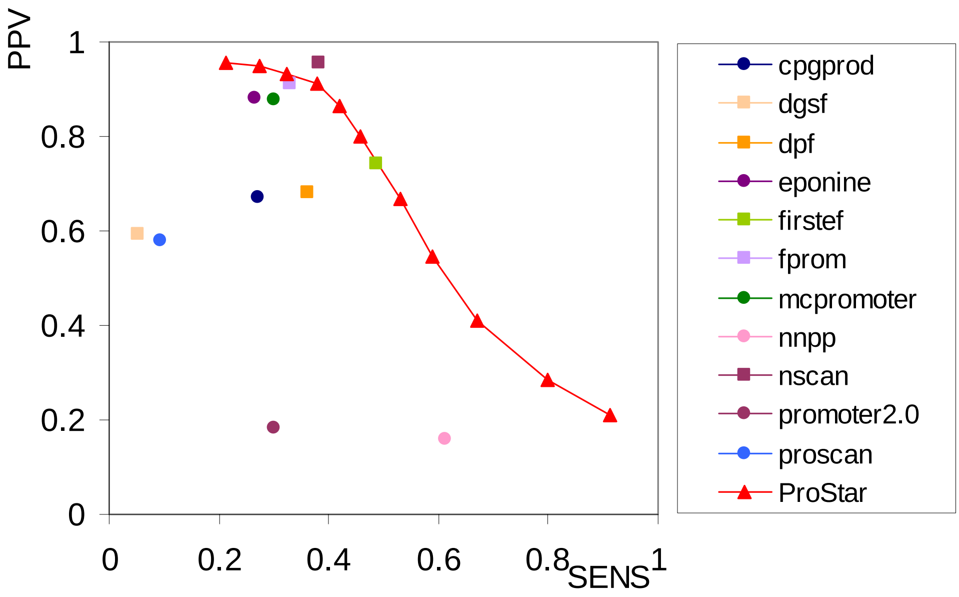

Figure S8. Test of ProStar behavior using those genes (2255) without TATA-box in the 5' end from the Havana set using tolerance D = 1000.

FIGURE S9

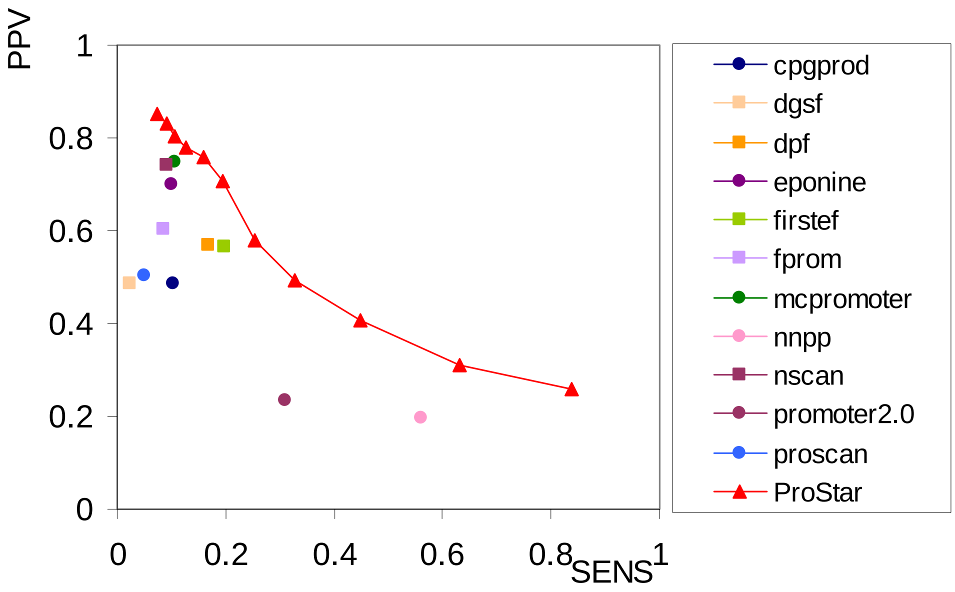

Figure S9. Test of ProStar behavior using CAGE TSS falling inside a transcribed region of any Havana gene (3270) using tolerance D = 1000.

FIGURE S9

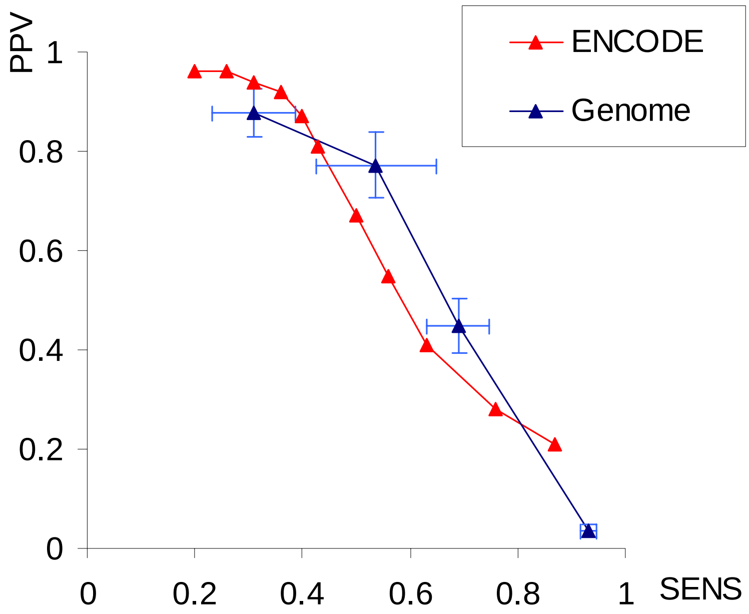

Figure S10. Comparing ProStar prediction power in Encode region (using Havana collection) and the whole human genome (using the RefSeq collection). Error bars are the standard deviation between chromosomes.
