# Supplementary material for: Determining promoter location based on DNA structure first-principles calculations
Source: Genome Biol. 2007 Dec 11;8(12):R263. doi: 10.1186/gb-2007-8-12-r263 (PMC2246265; doi:10.1186/gb-2007-8-12-r263)
Supplement: Additional file 2 — Promoter prediction methods described in this paper and a detailed evaluation of their performance. [file gb-2007-8-12-r263-S2.pdf]

Program name	Prediction source	Configuration	Homl	Gene	CpG	TATA
McPromoter	EGASP 7_80_8_ohler_mcpromoter_1113784666 [a]<br>EGASP 7_81_8_ohler_mcpromoter_noshadows_1113785822 [a]	standard; no shadows	No	No	No	Yes
N-SCAN	EGASP 20_76_4_multiple_v1_1113763810 [a]	standard	Yes	Yes	No	No
Fprom	EGASP 41_108_8_encode_promoters_1114770211 [a]	standard	No	Yes	Yes	Yes
CpGProD	http://pbil.univ-lyon1.fr/software/cpgprod_query.html [b]	default	No	No	Yes	No
DGSF	http://research.i2r.a-star.edu.sg/promoter/dragonGSF1_0/genestart.htm [b]	default	No	Yes	Yes	No
DPF	http://research.i2r.a-star.edu.sg/promoter/promoter1_5/DPF.htm [b]	0.5;0.55;0.65	No	Yes	Yes	No
EPONINE	http://www.sanger.ac.uk/Users/td2/eponine/eponine-scan.jar [c]	default	No	No	Yes	Yes
PROMOTER2.0	http://www.cbs.dtu.dk/services/Promoter/ [b]	default	No	No	No	No
PROSCAN	http://thr.cit.nih.gov/molbio/proscan/ [b]	default, 0.8	No	No	No	Yes
FirstEF	http://rulai.cshl.edu/tools/FirstEF [b]	default	No	Yes	Yes	No
NNPP	http://www.fruitfly.org/seq_tools/promoter.html [b]	0.8;0.9;0.95	No	No	No	Yes

Table S1. Sources of TSS predictions from other published methods. [a] Predictions are directly downloaded from EGASP ftp directory ftp://genome.imim.es/pub/projects/gencode/data/egasp05/*egasp_submissions_2005050*. [b] Predictions have been computed using the appropriate web resource. [c] Program has been download and executed to compute predictions. Columns 4, 5, 6 and 7 columns indicate if the method is based on conservation of DNA across the species, on gene structure, CpG content and TATA-box respectively.

ALL	SENS	PPV	SPEC	CC	AE	K2	Q	GDIP1	GDIP2	GDIP3	ASM
Prostar	0.33	0.78	0.99999	0.51	95.84	22189.18	0.99998	3.98E-05	2.01	3.98E-05	2.78
cpgprod	0.24	0.33	0.99997	0.28	115.17	13534.01	0.99982	5.34E-05	3.70	5.34E-05	8.33
dgsf	0.04	0.28	0.99999	0.10	133.25	16012.04	0.99971	5.74E-05	27.67	5.74E-05	9.11
dpf	0.27	0.38	0.99997	0.32	88.85	14379.95	0.99986	5.06E-05	3.14	5.06E-05	7.22
eponine	0.27	0.58	0.99999	0.40	93.66	18260.52	0.99994	4.46E-05	2.77	4.46E-05	5.22
firstef	0.46	0.42	0.99996	0.44	102.55	14491.43	0.99991	4.89E-05	1.81	4.89E-05	5.44
fprom	0.34	0.73	0.99999	0.50	58.07	21518.69	0.99997	3.97E-05	1.97	3.97E-05	2.89
mcpromoter	0.24	0.66	0.99999	0.40	69.76	19124.55	0.99995	4.54E-05	3.16	4.54E-05	5.33
nnpp	0.28	0.04	0.99955	0.10	101.24	2038.02	0.99765	4.50E-04	27.56	4.50E-04	10.89
nscan	0.38	0.82	1.00000	0.56	107.62	24160.85	0.99998	3.68E-05	1.62	3.68E-05	1.11
promoter2.0	0.13	0.04	0.99982	0.07	104.83	4253.96	0.99764	1.91E-04	23.98	1.91E-04	11.00
proscan	0.09	0.32	0.99999	0.17	112.36	15433.66	0.99977	5.51E-05	10.85	5.51E-05	8.67
TEST	SENS	PPV	SPEC	CC	AE	K2	Q	GDIP1	GDIP2	GDIP3	ASM
Prostar	0.29	0.76	0.99999	0.47	97.34	21436.38	0.99997	4.16E-05	2.43	4.16E-05	3.00
cpgprod	0.25	0.30	0.99997	0.27	110.31	12921.28	0.99980	5.52E-05	3.85	5.52E-05	8.44
dgsf	0.04	0.31	0.99999	0.11	127.27	16343.71	0.99975	5.63E-05	24.93	5.63E-05	8.78
dpf	0.24	0.36	0.99997	0.29	90.64	14422.16	0.99984	5.09E-05	3.62	5.09E-05	7.22
eponine	0.21	0.49	0.99999	0.32	100.51	17027.02	0.99991	4.77E-05	3.85	4.77E-05	6.11
firstef	0.40	0.40	0.99997	0.40	99.04	14422.16	0.99990	4.90E-05	2.11	4.90E-05	5.56
fprom	0.34	0.74	0.99999	0.50	62.39	21944.59	0.99997	3.92E-05	1.99	3.92E-05	2.67
mcpromoter	0.23	0.68	0.99999	0.39	81.53	19468.89	0.99996	4.54E-05	3.40	4.54E-05	4.67
nnpp	0.27	0.03	0.99955	0.10	97.43	2041.68	0.99762	4.49E-04	28.23	4.49E-04	11.11
nscan	0.35	0.82	1.00000	0.54	103.82	23624.88	0.99998	3.82E-05	1.87	3.82E-05	1.11
promoter2.0	0.14	0.04	0.99982	0.08	100.91	4333.93	0.99775	1.87E-04	23.26	1.87E-04	10.89
proscan	0.08	0.30	0.99999	0.15	114.03	15568.96	0.99975	5.48E-05	12.13	5.48E-05	8.44

Table S2. Prediction results using the Havana annotations over the Encode region. Table shows scores for the training set and the test set using a maximum distance for TP of 250nt (D = 250). Measures are described in Supplementary Methods.
