# Supplementary material for: Determining promoter location based on DNA structure first-principles calculations
Source: Genome Biol. 2007 Dec 11;8(12):R263. doi: 10.1186/gb-2007-8-12-r263 (PMC2246265; doi:10.1186/gb-2007-8-12-r263)
Supplement: Additional file 3 — Extended description of the performance test and the averaged score measure. [file gb-2007-8-12-r263-S3.pdf]

Supplementary Methods

The following measures describes program performances and do not strongly correlate between them (Bajic et al. 2000). Here TP is the number of true positive, TN the number of true negatives, FN the number of false positives FP the number of false positives and N_{tot} = TP+ TN+ FP+ FN.

Sensitivity (SENS)

$$SENS = \frac{TP}{TP + FN}$$

Specificity (SPEC)

$$SPEC = \frac{TN}{TN + FP}$$

Second specificity coefficient or proportion of correct predictions (PPV)

$$PPV = \frac{TP}{TP + FP}$$

Second Prediction Quality Coefficient (K2)

$$K2 = \frac{TP + TN}{FN + FP + \frac{1}{N_{tot}}}$$

Yules association coefficient (Q)

$$Q = \frac{(TP \times TN) - (FP \times FN)}{(TP \times TN) + (TP \times TN)}$$

Generalized distances from the ideal predictor (GDIP1, GDIP2, GDIP3)

$$GDIP1 = \frac{\sqrt{FP^2 + FN^2}}{TP + TN + \frac{1}{N_{tot}}}$$

$$GDIP1 = \frac{\sqrt{FP^2 + FN^2}}{TP + \frac{1}{N_{tot}}}$$

$$GDIP1 = \frac{\sqrt{FP^2 + FN^2}}{TN + \frac{1}{N_{tot}}}$$

Averaged score mesure (ASM)

Assuming that we used z measures to rank predictor scores of p programs, we define for the ith method the row vector $r_i = [P^i_1, \ldots, P^i_z]$. Then we compute the ASM score of those methods as:

$$ASM_i = \frac{1}{z}\sum_{j=1}^{z} P_j^i$$

Following Bajic's paper (Bajic et al. 2000), we used the following non-correlated measures for ASM calculation: Q, K2, CC, GDIP1, GDIP2, GDIP3, SENS, PPV and SPEC. ASM is a global measure of the relative quality of a predictive method with respect to alternative approaches (Bajic et al. 2000).
